# Cyclic-di-AMP confers an invasive phenotype on *Escherichia coli* through elongation of flagellin filaments

**DOI:** 10.1186/s13099-024-00600-4

**Published:** 2024-01-24

**Authors:** Rika Tanaka, Jin Imai, Eiji Sugiyama, Shogo Tsubaki, Katsuto Hozumi, Hitoshi Tsugawa

**Affiliations:** 1 Department of Immunology, Division of Host Defense Mechanism, Tokai University School of Medicine, Isehara, Japan; 2 Department of Clinical Health Science, Tokai University School of Medicine, Isehara, Japan; 3 Laboratory of Analytical and Bio-Analytical Chemistry, School of Pharmaceutical Sciences, University of Shizuoka, Shizuoka, Japan; 4 Transkingdom Signaling Research Unit, Division of Host Defense Mechanism, Tokai University School of Medicine, 143 Shimokasuya, Isehara, Kanagawa 259-1193 Japan

**Keywords:** Adherent-invasive *Escherichia coli* (AIEC), Cyclic di-adenosine monophosphate (c-di-AMP), Crohn’s disease, Flagellin filament, Inflammatory bowel disease (IBD), Intestinal epithelium

## Abstract

**Background:**

Adherent-invasive *Escherichia coli* (AIEC) is isolated from patients with Crohn’s disease (CD). AIEC can invade the intestinal epithelium, suggesting that it is involved in the development and pathogenesis of CD. However, the mechanism by which AIEC acquired the invasive phenotype remains unknown.

**Results:**

This study was designed to examine the mechanisms of AIEC invasiveness. We found that the flagellin (*fliC*) expression in AIEC was two-fold higher than that in non-AIEC strains, and this overexpression induced the formation of long-filament flagellin. Deletion of *fliC* in the AIEC LF82 strain resulted in the disappearance of flagellar filaments and attenuated the motility and invasive ability of the bacterium, suggesting that the formation of long filament flagellin induced by increased *fliC* expression is required by AIEC to invade the intestinal epithelium. In AIEC and non-AIEC K12 strains cultured in the presence of cyclic-di-AMP (c-di-AMP), the expression of *fliC* was enhanced, and flagellar filaments were elongated. Stimulation with c-di-AMP enhanced the bacterial motility and ability to invade epithelial cells, even in the non-AIEC K12 strain.

**Conclusions:**

Our findings show that c-di-AMP confers an AIEC-like phenotype on non-AIEC strains by enhancing the expression of *fliC*. The results should be useful for understanding the pathogenesis of CD.

**Supplementary Information:**

The online version contains supplementary material available at 10.1186/s13099-024-00600-4.

## Background

Crohn’s disease (CD) is an inflammatory bowel disease (IBD), with courses of ulceration, inflammation, and fibrosis in the intestine and digestive tract. CD is an intractable disease of unknown etiology that is more common among young people. The incidence of IBD is increasing worldwide, particularly in the developed countries [1, 2]. From several previous studies have shown alterations in the gut microbiota of patients with CD compared to healthy subjects, suggesting that dysbiosis of the gut microbiota is associated with the development and pathogenesis of CD [3, 4].

Commensal gut bacteria communicate with each other to coordinate their behavior and function. Bacterial communication is achieved by modifying gene expression using second messengers. Cyclic di-AMP (c-di-AMP), a cyclic nucleotide produced by bacteria, is a second messenger that regulates bacterial gene expression by binding to its target effectors.

Adherent-invasive Escherichia coli (AIEC) have been isolated from the distal end of the ileum, a common stricture site in patients with CD [5]. Unlike non-AIECs, AIECs adhere and invade the intestinal epithelium. The adhesion of AIEC is mediated by interactions between bacterial type-1 pili and carcinoembryonic antigen-related adhesion molecule 6 (CECAM6) on the surface of the host epithelium [6]. The bacterial invasion is promoted by the binding of bacterial outer membrane proteins (*OmpA*) to the ER-stress response chaperone, Gp96, which is strongly expressed on the apical surface of ileal epithelial cells [7, 8]. The specific adhesion and invasiveness of AIEC are believed to strongly contribute to the development and pathogenesis of CD, especially the postoperative recurrence [9].

The flagellum of E. coli is a complex structure consisting of three main structural regions: the basal body, hook, and filament. The flagellin filament extends outward from the basal body and is a polymer of the flagellin subunits encoded by *fliC*. Flagellin filaments are responsible for the ability of AIEC strains to cross the mucus layer in vitro and in vivo and reach the epithelial surface [10].

We recently showed that AIEC-specific IgA antibodies are produced in the gastrointestinal tract of AIEC-infected mice. The IgA antibody specific to AIEC could inhibit its ability to adhere to and invade the intestinal epithelial Caco-2 cells [11]. However, it remains unclear how AIEC acquired the ability to adhere to and invade the intestinal epithelium. The present study was aimed at elucidating the acquisition mechanisms of adhesion and invasiveness of AIEC. The results implicate the role of flagellin elongation in *E. coli* mediated through c-di-AMP in the invasive ability of AIEC. The results of this study should help better understand the pathogenesis of CD and aid in devising better therapeutic strategies against this intractable disease.

## Results

### Flagellar filaments of AIEC are elongated as a result of increased fliC expression.

**Fig. 1:**
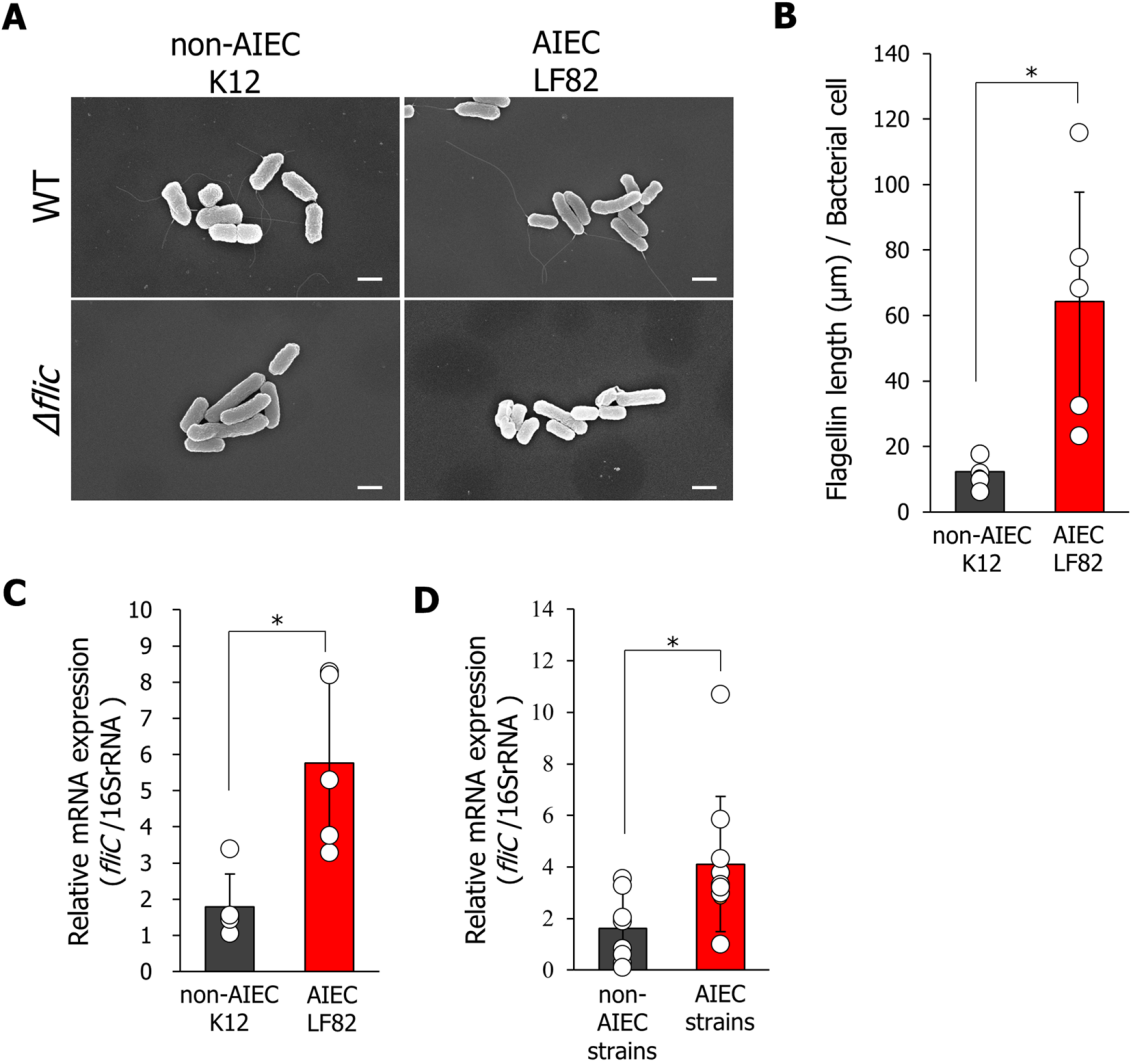
Flagellin in the AIEC strains is elongated depending on the increase in *fliC* expression. **A** Morphological changes in non-AIEC K12 and AIEC LF82 strains were visualized using SEM. Each image represents a different bacterial area. Scale bar = 1.0 μm. **B** Flagellin length per bacterial cell was measured using the ImageJ software. *P < 0.05; *P*-values were determined using the unpaired Student’s *t*-test. **C** Comparison of *fliC* mRNA expression levels between AIEC and non-AIEC strains clinically isolated from patients with Crohn’s Disease (CD) was performed using quantitative real-time RT-PCR. Data are presented as the mean ± SD. *P < 0.05; *P*-values were determined using the unpaired Student’s *t*-test. (*n* = 10 strains each). **D** Comparison of *fliC* mRNA expression levels between the AIEC LF82 and non-AIEC K12 strains. Data are presented as the mean ± SD. *P < 0.05; *P*-values were determined using the unpaired Student’s *t*-test. (*n* = 3 for each strain)

Bacterial flagella mediate not only motility but also bacterial adhesion and invasion [12, 13]. We compared the flagellar structure in the AIEC LF82 strain isolated from patients with CD with that of *E. coli* K12 strain as a non-AIEC reference strain. The flagellar length of the AIEC LF82 strain was significantly greater than that of the non-AIEC K12 strain (Fig. 1a, b). The flagellar structure in *E. coli* contains three distinct components: a basal body, hook, and extracellular filament. The flagellin (FliC) subunit is the main component of the flagellar structure [14]. To clarify the involvement of FliC in flagellar elongation in the AIEC LF82 strain, *fliC* deletion mutants (*ΔfliC* strains) were constructed using homologous recombination (Additional file 1: Fig. S1a). We confirmed the deletion of *fliC* gene (1489 bp) using polymerase chain reaction (PCR) (Additional file 1: Fig. S1b). The flagellar filaments of both the AIEC LF82 and non-AIEC K12 strains completely disappeared after *fliC* deletion (Fig. 1a). We then compared the expression levels of *fliC* mRNA in AIEC LF82 with those in the non-AIEC K12 strain. *fliC* expression levels in the AIEC LF82 strain were significantly higher than those in the non-AIEC K-12 strains (Fig. 1c). Additionally, the levels of *fliC* mRNA expression were enhanced in several AIEC strains compared with those in several non-AIEC strains, which indicates that increased *fliC* expression inducing flagellar elongation is a characteristic of the AIEC strain (Fig. 1d).

### Enhanced bacterial migration and invasive abilities of AIEC are provided by increased fliC expression.

**Fig. 2:**
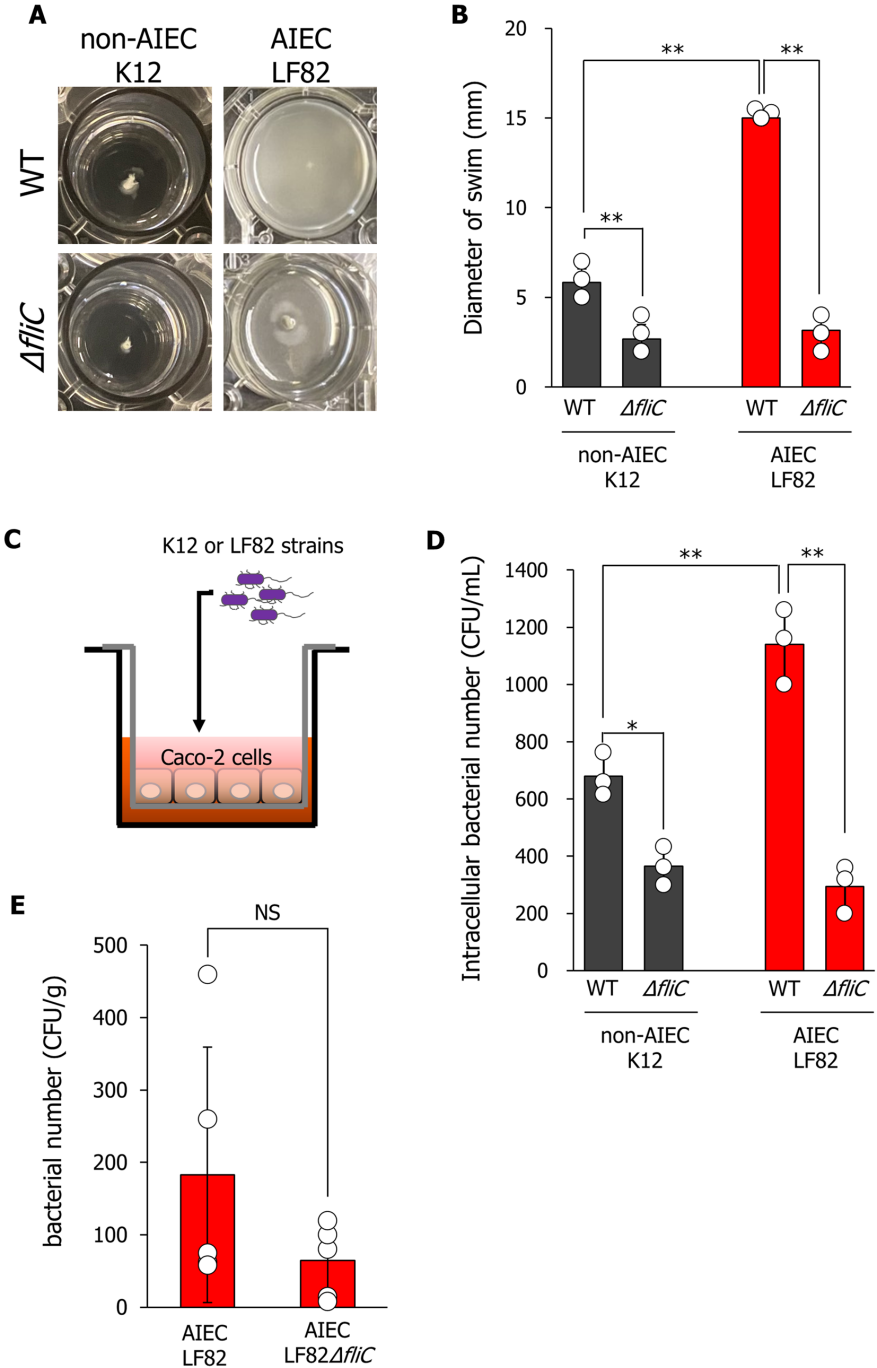
Migration and invasive abilities of the AIEC LF82 strain are promoted depending on the increase in *fliC* expression. **A** Representative images of the bacterial growth zones (swimming diameters) for non-AIEC K12 and AIEC LF82 strains. Each bacterial strain (OD_570_ = 1.0) was added to the center of a plate containing Luria Bertani (LB) medium containing 0.3% agar and incubated at 37 °C for 16 h. **B** Bacterial swimming diameters on LB agar plates were measured using the ImageJ software. Data are presented as mean ± SD. *P < 0.05, **P < 0.01; *P*-values were determined using the student’s *t*-test. **C** In vitro infection model using Transwell inserts. Caco-2 cells grown on the insert were incubated with the bacteria at a multiplicity of infection (MOI) of 1:100 (1 × 10^5^ bacteria/well) for 30 min. Caco-2 cells grown on the inserts were incubated with the bacteria for 3 h and then washed and incubated for 3 h in Dulbecco’s modified Eagle medium (DMEM) containing 400 μg/mL kanamycin to kill extracellular bacteria. **D** Caco-2 cells were lysed with PBS containing 1% Triton X-100, and the lysates were plated on LB agar. Colony-forming units (CFU) were counted after 24 h of incubation. Data are presented as the mean ± SD. *P < 0.05, **P < 0.01; *P*-values were determined using the student’s *t*-test. **E** Bacterial counts in the colon were determined five days after infection. Mice were administered antibiotics (20 mg/L streptomycin and 1 g/L ampicillin) for 9 days and then orally inoculated with 1 × 10.^9^ CFU of LF82 and *ΔfliC-*LF82. The colon tissue was homogenized in PBS, and the homogenates were plated onto MacConkey agar containing 50 μg/mL ampicillin. The number of CFU was counted. Data are presented as mean ± SD (*n* = 5 per group)

To examine whether the migration ability of the AIEC LF82 strain was enhanced, the swimming motility of the bacterium was measured using Luria Bertani (LB) medium containing 0.3% agar [9]. The swimming diameters of the AIEC LF82 strain were significantly greater than those of the non-AIEC K12 strain (Fig. 2a, b). The enhanced swimming ability of the AIEC LF82 strain was significantly attenuated to the same level as that of the non-AIEC K12 strains by deletion of *fliC* (Fig. 2b). Next, we constructed in vitro bacterial infection models using Caco-2 cells grown on Transwell inserts to evaluate the bacterial invasion ability (Fig. 2c). Caco-2 cells grown on Transwell inserts were infected with each bacterium (1 × 10^5^ bacteria) for 3 h and then incubated for 3 h with Dulbecco's modified Eagle medium (DMEM) containing kanamycin to kill extracellular bacteria. The number of bacteria within Caco-2 cells infected with the AIEC LF82 strain was significantly higher than that in cells infected with the non-AIEC K12 strain (Fig. 2d). The *fliC* deletion significantly decreased the number of invading bacteria in both non-AIEC K12 and AIEC LF82 strains and attenuated the enhanced invasive ability of the AIEC LF82 strain compared with that of the non-AIEC K12 strain (Fig. 2d). Additionally, in AIEC LF82-infected mice, the number of invasive bacteria in the cecal epithelium was decreased by deletion of *fliC* (Fig. 2e). Together, our results show that the bacterial migration and invasion abilities of the AIEC LF82 strain were enhanced by the increased *fliC* expression.

### c-di-AMP elongates flagella in both non-AIEC K12 and AIEC LF82 strains by enhancing fliC expression.

**Fig. 3:**
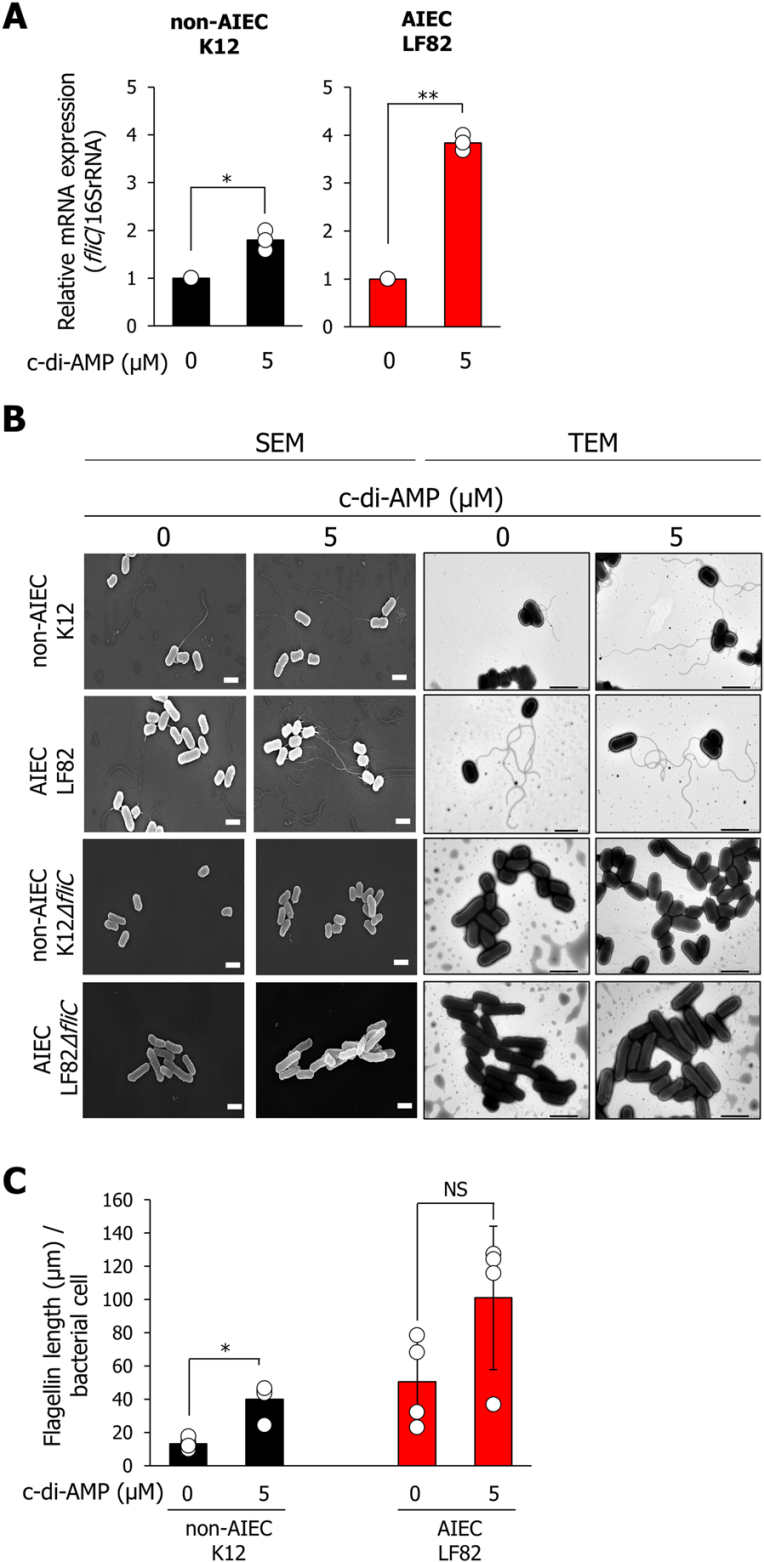
C-di-AMP enhances the expression of *fliC* and elongates the flagellin in both non-AIEC K12 and AIEC LF82 strains. **A** The expression levels of *fliC* in the non-AIEC K12 and AIEC LF82 strains, which were cultured in the presence of 5.0 μM c-di-AMP for 16 h, were analyzed using quantitative real-time PCR. Data are presented as the mean ± SD. *P < 0.05, **P < 0.01; *P*-values were determined using the unpaired Student’s *t*-test. (*n* = 4 each). **B** Morphological changes in non-AIEC K12 and AIEC LF82 strains cultured in the presence of 5.0 μM c-di-AMP for 16 h were visualized using scanning electron microscopy (SEM). Each image is representative of different bacterial areas (*n* = 4 each). **C** Length of flagellin filaments measured using SEM images. SEM images were imported into the ImageJ software, and the flagellin length per bacterial cell was measured. Data are presented as the mean ± SD. *P < 0.05; *P*-values were determined using the student’s *t*-test. Scale bar = 2.0 μm

c-di-AMP is a bacterial second messenger that regulates the expression of several genes involved in DNA damage, biofilm formation, acid stress resistance, and other functions [15–19]. We hypothesized that the expression of *fliC* in *E. coli* is enhanced by c-di-AMP signaling. To test this hypothesis, we examined changes in *fliC* expression and flagellar elongation in the presence of c-di-AMP. The expression of *fliC* in both non-AIEC K12 and AIEC LF82 strains increased 2- and fourfold, respectively, in the presence of 5.0 μM c-di-AMP (Fig. 3a). Next, we examined the flagellar length in each bacterium cultured in the presence of c-di-AMP using scanning electron microscopy (SEM) and transmission electron microscopy (TEM). Additionally, we calculated the flagellar length using SEM analysis data, because SEM can efficiently image the whole bacterial structure and has been used for observations of the bacterial cell surface contaminations and biofilm structure [20, 21]. The results showed that, in the presence of c-di-AMP, the flagellar length is increased in both non-AIEC K12 and AIEC LF82 strains (Fig. 3b, c). In *fliC*-deletion mutant strains, elongated flagellar structures were not observed even in the presence of c-di-AMP (Fig. 3b).

### c-di-AMP confers an AIEC-like phenotype to non-AIEC strains by enhancing fliC expression.

**Fig. 4:**
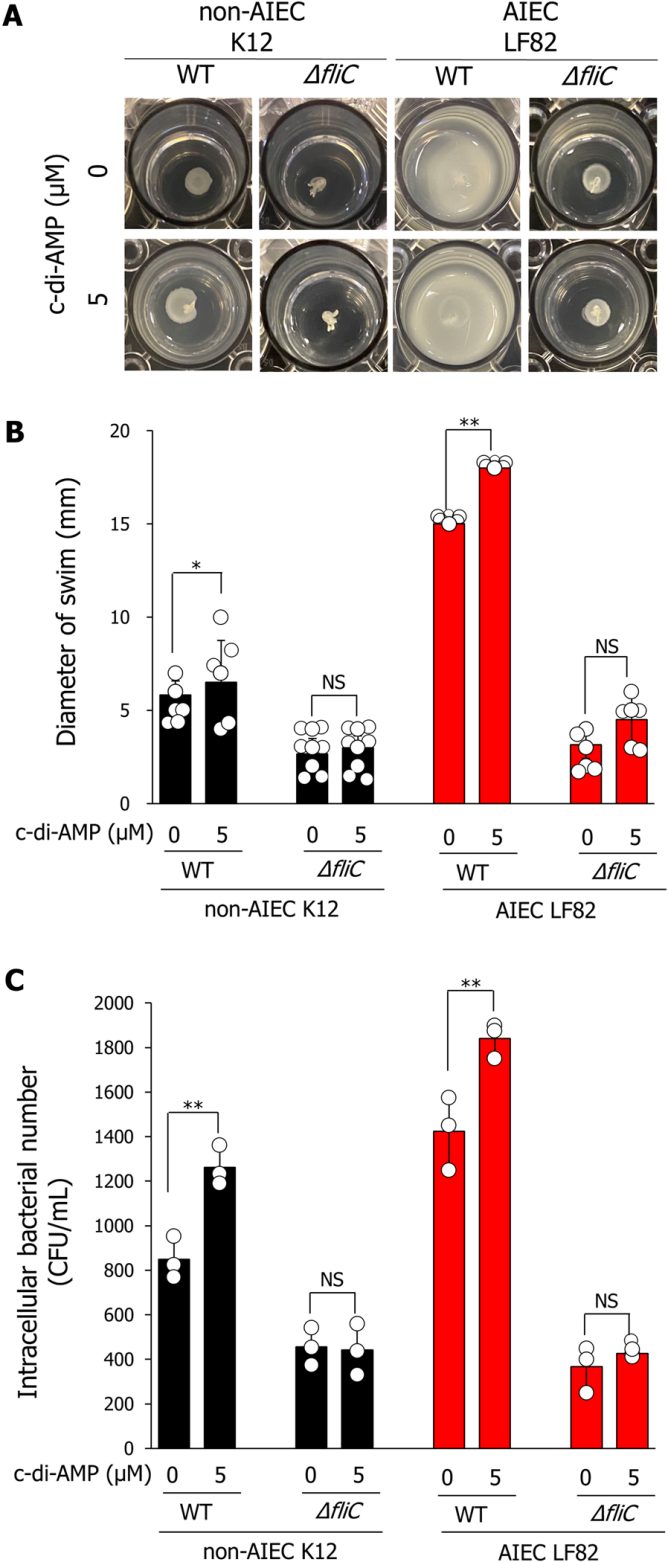
C-di-AMP enhances migration and invasion abilities of both non-AIEC K12 and AIEC LF82 strains. **A** Representative images of the bacterial growth zone (swimming diameters) of the non-AIEC K12 and AIEC LF82 strains in the presence of 5.0 μM c-di-AMP. Each bacterial strain (OD_570_ = 1.0) was added to the center of a plate containing Luria Bertani (LB) medium containing 0.3% agar and 5.0 μM c-di-AMP, and then the plate was incubated at 37 °C for 16 h. **B** Bacterial swimming diameters on LB agar plates were measured using the ImageJ software. Data are presented as mean ± SD. *P < 0.05, **P < 0.01; *P*-values were determined using the student’s *t*-test. **C** Each bacterial strain was precultured in LB broth containing 5.0 μM c-di-AMP for 16 h, and then added to Caco2 cells at a multiplicity of infection (MOI) of 1:100 (1 × 10^5^ bacteria/well). After 3 h incubation, each bacterial strain was added to Caco-2 cells (MOI of 1:100 (1 × 10.^5^ bacteria/well) for 3 h and incubated for 3 h in DMEM containing 400 μg/mL kanamycin to kill extracellular bacteria. The Caco2 cells were lysed with PBS containing 1% Triton X-100, and the lysates were plated on LB agar. Colony-forming units (CFU) were counted after 24 h of incubation. Data are presented as the mean ± SD. **P < 0.01; *P*-values were determined using the student’s *t*-test. (*n* = 3)

We examined whether the flagellar elongation induced by c-di-AMP affected bacterial migration and invasion. The swimming diameters of both the non-AIEC K12 and AIEC LF82 strains were significantly extended in the presence of c-di-AMP, but not in the *fliC*-deletion mutant strain (Fig. 4a, b). Additionally, the numbers of intracellular bacteria within Caco-2 cells infected with the non-AIEC K12 and AIEC LF82 strains were significantly increased in the presence of c-di-AMP (Fig. 4c). In contrast, in *fliC*-deletion mutant strain-infected Caco-2 cells, c-di-AMP stimulation did not increase the number of intracellular bacteria (Fig. 4c). These findings suggest that c-di-AMP enhances bacterial motility and invasiveness through the elongation of flagella by increasing the expression of *fliC* (Fig. 4c).

c-di-AMP is synthesized and secreted by several types of bacteria, mainly gram-positive bacteria [19]. We hypothesized that intracellular c-di-AMP synthesis is specifically enhanced in the AIEC strain compared with that in the non-AIEC strains. To prove this, we measured the c-di-AMP concentration within bacterial cells through liquid chromatography-tandem mass spectrometry. However, intracellular c-di-AMP was not detected in either the AIEC or non-AIEC strains, suggesting that c-di-AMP is not synthesized even in AIEC strain (Additional file 1: Fig. S2a). This may be because *E. coli*, which belongs to the class *Deltaproteobacteria* does not encode the diadenylate cyclase domain, which converts two ATP or ADP molecules into one c-di-AMP molecule [19, 22]. From these observations, we can assume that *E. coli* accepts extracellular c-di-AMP produced by other gut microbial communities.

## Discussion

AIEC strains are capable of adhering to and invading the intestinal epithelium, unlike the intestinal commensal *E. coli* [23]. Although AIEC strains are thought to contribute to the development of CD, the origin and/or route of infection is not well known. Alterations in the composition of the gut microbiota in patients with CD compared with that in healthy donors has been reported [24]. Imbalances in the gut microbiota affect the overall structure of the microbiota community and specific bacterial expansion by altering bacterial cell–cell communication [25]. In the present study, we examined whether c-di-AMP, a bacterial cell–cell communication messenger, affects the *E. coli* phenotype associated with invasion of the host epithelium. The results showed that c-di-AMP enhanced the motility of bacteria and their ability to invade epithelial cells, in both AIEC non-AIEC strains, by elongating the bacterial flagellum. Our findings suggest that c-di-AMP confers an AIEC-like phenotype on non-AIEC strains in the gut lumen.

Genes involved in the flagellar assembly are classified into three groups: classes I–III [26]. Class III genes are involved in fiber formation, the final step in flagellar development (including flagellar fiber-forming flagellin), and in the functioning of the completed flagellum [26]. The *fliC* genes belonging to class III encode flagellin, which forms flagellar fibers. Genes belonging to class III are downstream of class II genes; therefore, the expression of class III genes is regulated by class II promoters. Flagella are involved in the ability of AIEC to cross the mucus layer; therefore, enhancement of bacterial motility by the flagellar function is believed to confer a selective advantage to *E. coli* in penetrating the mucus layer and reaching the epithelial surface [23]. In AIEC infection models in vivo, the deletion of *fliC* decreased bacterial adhesion to the intestinal epithelium (Fig. 2e). Additionally, the deletion of *fliC* significantly decreased bacterial motility in both the AIEC and non-AIEC strains (Fig. 2a, b). These findings suggest that the acquisition of enhanced bacterial motility by increasing the expression of *fliC* hastens the approach of the bacteria to the epithelial surface, leading to enhanced adherence and invasiveness. The results showed that the *fliC* expression levels regulate the length of flagellar filaments, contributing to bacterial invasiveness.

In the present study, we showed that c-di-AMP enhances the flagellin expression, motility and invasiveness of *E. coli,* supported by the analysis of both non-AIEC K12 and AIEC LF82 strains. To date, as shown in Fig. 1d, several AIEC and non-AIEC strains have been clinically isolated from patients with CD. However, the whole genome information of these clinically isolated strains has not been published. Therefore, it is not possible to construct a *fliC*-deletion mutant strain, and the genetic diversity of each strain involved in c-di-AMP responses is difficult to understand. AIEC LF82 has been isolated from patients with CD and is the representative strain of AIEC [27]. The non-AIEC K12 strain has been used in many experiments as the representative strain of *E. coli* [28, 29]. The whole genome sequences are available for both of these strains (K12 strain: NCBI Reference Sequence: U00096.3, LF82 strain NCBI Reference Sequence: NC_011993.1). Therefore, we could construct the *fliC*-deletion mutant strains. Given the reasons we explain above, increasing the number of bacterial strains for analysis is difficult, and this was an experimental limitation of our study. Although the present study showed that the c-di-AMP conferred AIEC-like phenotypes to non-AIEC strains by enhancing flagellin expression, motility and invasiveness, as well as elongating flagellum, further studies that include larger sample size of clinically isolated non-AIEC and AIEC strains are needed.

As evident from Additional file 1: Figure S2, neither the AIEC nor the K12 strain synthesizes c-di-AMP, and thus we speculate that extracellular c-di-AMP released by other gut microbiota communities is sensed and utilized. The composition of the gut microbiota is known to be altered in patients with CD compared with that in healthy donors; therefore, we assume that the gut microbial composition is enriched with microorganisms possessing high c-di-AMP production abilities in patients with CD. Interestingly, several gram-positive bacteria have been identified as high c-di-AMP producers and a relatively high proportion of gram-positive bacteria has also been reported in the gut microbiota of patients with IBD [30, 31]. Hence, the production of c-di-AMP in the gastrointestinal lumen of patients with IBD may be elevated. Although further studies are needed to identify the gut microbiota communities that enhance c-di-AMP levels in patients with CD, our findings reveal that extracellular stimulation of c-di-AMP in the gut lumen induces the expression of *fliC* and confers the invasiveness of *E. coli*.

## Conclusion

AIEC isolated from patients with CD have the ability to adhere and invade the intestinal epithelium and are involved in the development and recurrence of CD. However, it remains unknown how exactly AIEC acquire these abilities. The aim of this study was to examine the acquisition mechanism of adhesion and invasiveness in *E. coli*. Here, we show that flagellin gene expression in clinically isolated AIEC strains is increased by two-fold compared to that of non-AIEC strains, and the deletion of *fliC* in AIEC strains attenuates bacterial motility and adhesion ability. The expression of *fliC* in non-AIEC strains was increased by treatment with cyclic-di-AMP, resulting in the formation of long-filament flagellin. Furthermore, stimulation by cyclic-di-AMP enhanced bacterial motility and the ability to adhere to epithelial cells in the non-AIEC strain. Our findings demonstrated that cyclic-di-AMP confers an AIEC-like phenotype to non-AIEC strains via the enhancement of *fliC* expression.

## Methods

### Ethics for human studies

The study design was reviewed and approved by the Institutional Review Board of Tokai University School of Medicine (No. 20I-35) and was conducted according to principles of the Declaration of Helsinki. Written informed consent was obtained from all participants before their inclusion in the study.

### Ethics for animal studies

Animal experiments were approved by the Tokai University (Kanagawa, Japan) Animal Research Committee (No. 220244) and were conducted in accordance with the “Act on Welfare and Management of Animals of Japan,” “Standards relating to the Care and Keeping and Reducing Pain of Laboratory Animals,” “Standards relating to the Methods of Destruction of Animals,” “Guidelines for Proper Conduct of Animal Experiments,” and “Fundamental Guidelines for Proper Conduct of Animal Experiments.”

### Bacterial strains and cell lines

*Escherichia coli* K12 ATCC10798, purchased from the American Type Culture Collection (ATCC), was used as the non-AIEC strain. *E. coli* LF82 strain was isolated from a chronic ileal lesion in a patient with CD and used as the AIEC strain [32]. The following clinical isolates of *E. coli* were used in this study: *E. coli* HS, LF1, LF6, LF19, LF48, LF55, LF111, LF134, LF135, LF82, LF16, LF31, LF73, 6076, 6088, 6170, 6254, 6259, and 6283 [33, 34]. All bacteria were routinely cultured in LB broth (Nacalai Tesque Inc., Japan) overnight at 37 °C with shaking at 160 rpm until their growth reached the mid-exponential phase (OD_570_ = 1.0).

Human colon adenocarcinoma, Caco-2, cells were purchased from the European Collection of Cell Cultures (ECACC 86010202). Caco-2 cells were maintained in a 5% CO_2_ atmosphere at 37 °C in DMEM (Gibco), supplemented with 10% (v/v) fetal bovine serum (Gibco), 0.01% minimum essential medium non-essential amino acids solution (Invitrogen), 0.01% L-glutamine (Invitrogen), and 0.01% penicillin–streptomycin (Nacalai Tesque Inc.).

### Construction of fliC deletion mutant

The target-region gene cassette (5′*fliC*–*ampicillin resistance gene*–3′*fliC*) for construction of a *fliC*-deletion mutant was cloned into a temperature sensitive pTSC30 plasmid [35]. The cassette was inserted into the open reading frame of *fliC*. The target-region gene cassette was constructed using the PCR-based overlap extension method. The primer sequences used for the construction of the cassette were as follows: 5′*flic* region: forward 5′-CTCGAGCATGGCACAAGTCAT and reverse 5′-CGACACGGAAATGTTGAATACTCATAATTTCGTCCTGGATAGAAGACAG; *ampicillin resistance gene region: forward* 5′-CTGTCTTCTATCCAGGACGAAATTATGAGTATTCAACATTTCCGTGTCG and reverse 5′-CTACAGATGCGATAGCATCGTCCAGACCAATGCTTAATCAGTGAGGCAC; 3′*flic* region: forward 5′-GTGCCTCACTGATTAAGCATTGGTCTGGACGATGCTATCGCATCTGTAG and reverse 5′-GGATCCTTAACCCTGCAGCA. For overlap extension, the forward primer used was the 5′*flic* region forward primer and the reverse primer was the 3′*flic* region reverse primer. The target-region gene cassette was inserted into the pTSC30 plasmid using the XhoI and BamHI sites and NEBuilder HiFi DNA Assembly Master Mix (New England BioLabs). The target pTSC30 plasmid was electroporated into *E. coli* K12 cells, which were cultured overnight and selected on LB agar containing 50 μg/mL ampicillin at 37 °C. The AIEC LF82 *fliC* deletion mutant strain (AIEC LF82 *ΔfliC*) was a gift from Dr. Nobuhiko Kamada (Department of Pathology and Comprehensive Cancer Center, University of Michigan, USA) [36].

### In vivo* bacterial infection*

All animal experiments were approved by the Tokai University (Kanagawa, Japan) Animal Research Committee (No. 220244). Seven- to eight-week-old C57BL/6 J female mice were purchased from CLEA Japan Co., Ltd. The mice were provided drinking water containing 20 mg/L streptomycin and 1 g/L ampicillin for 1 week prior to bacterial infection. They were orally inoculated with 200 μL LB broth containing AIEC LF82 or LF82-*ΔfliC* (1 × 10^9^ bacteria) using a stainless-steel feeding needle fitted to a 1.0 mL syringe and sacrificed 5 days after infection. The colorectal tissue was harvested and homogenized using a Biomasher II (Nippi, Japan). Serial dilutions of the homogenates were plated on LB agar, and colony-forming units (CFUs) were counted after 24 h of incubation.

### In vitro* bacterial infection*

Caco-2 cells (2 × 10^5^ cells/well) were seeded in each well of 6-well Transwell insert culture plates (0.4 µm pore size) (Corning, Lowell) at day 7. Bacteria were precultured overnight with or without 5.0 µM c-di-AMP (Sigma) in LB broth. Caco-2 cells were infected with each bacterial strain (1 × 10^5^ bacteria) at a multiplicity of infection of 0.5 for 3 h and then incubated with DMEM containing kanamycin for 3 h to kill extracellular bacteria. The cells were lysed with PBS containing 1% Triton X-100 and the lysates were plated on LB agar. The number of CFUs was counted after 24 h incubation at 37 °C.

### Extraction of total RNA and quantitative real-time RT-PCR

AIEC and non-AIEC strains were preincubated overnight with or without 5 µM c-di-AMP (Sigma). Total RNA was isolated from bacteria using the SV Total RNA Isolation Kit (Promega) according to the manufacturer’s guidelines. The extracted total RNA was reverse-transcribed into single-stranded cDNA using the PrimeScript™ RT reagent kit (Takara). PCR was performed using the StepOne™ Real-Time PCR system with PowerTrack™ SYBR Green Master Mix (Thermo Fisher Scientific). The sequences of primers were as follows: non-AIEC: F: 5′-ATTCCGTTCTTCCCTCGGTG-3′, R: 5′-TGGACACTTCGGTCGCATAG-3′ (amplicon size: 131 bp) and F: 5′-ATGGCACAAGTCATTAATACC-3′, R: 5′-AAGACAGACGCTCGATAGAAC-3′ (amplicon size: 100 bp); AIEC: F: 5′-TGGTGCTGCAACTGCTAACGC-3′, R: 5′-TTATCGGCATATTTTGCGCTAGC-3′ (amplicon size: 212 bp) and F: 5′-CGGCAAATACCGCCTGATACG-3′, R: 5′-GCTACAGCTAAACAAGGCACA-3′ (amplicon size: 100 bp). Relative gene expression levels were measured using the 2nd Derivative Maximum method. The 16* s* rRNA gene was used as a quantitative loading control and was amplified using the following primers: F: 5′-CATGCCGCGTGTATGAAGAA-3′ and R: 5′-CGGGTAACGTCAATGAGCAAA-3′.

### Swimming motility assay

Bacterial swimming motility was evaluated in LB broth containing 0.3% agar (Becton Dickinson) and 0.3% glucose (Nacalai Tesque Inc.). Each bacterial strain was precultured with or without 5 µM c-di-AMP, and then 5 µL of the bacterial solution (OD_570_ = 1.0) was dropped onto the center of the LB medium [12]. After overnight culture, diameters of bacterial colonies were measured using the ImageJ software (National Institutes of Health).

### Scanning electron microscopy

Bacteria were centrifuged (11000 × g, 3 min, 4 °C) and fixed overnight in 1% glutaraldehyde prepared in 0.1 M phosphate buffer* (*pH 7.4*)* at 4 °C or for 30 min at 25–28 ℃ and washed three times with 0.2 M cacodylate buffer for 10 min each. The cells were then fixed by incubation for 1 h in 1%OsO_4_ − 0.1 M Phosphate Buffer (pH 7.4) and subsequently dehydrated using a graded series of ethanol (50%, 70%, 90%, 95%, and 100%). The cells were then washed (15 min for each wash), and dried. After dehydration using a graded acetone series, *E. coli* cells were transferred to tert-butyl alcohol and frozen. Frozen *E. coli* cells were freeze-dried in a VFD-30 freeze-drying device (Vacuum Device Ltd., Ibaraki, Japan) and coated with osmium using a Neoc-Pro osmium coater (Meiwafosis Co., Ltd., Tokyo, Japan). The SEM images were obtained using a JSM-6510LV scanning electron microscope (JEOL, Tokyo, Japan) at 15 kV. The length of flagellin filaments was measured using the ImageJ software (National Institutes of Health).

### Transmission electron microscopy

Bacteria were gently resuspended in TEM sample buffer with 1% glutaraldehyde in 0.1 M phosphate buffer (pH 7.4) overnight at 4 °C. A 10 μL sample was pipetted onto Formvar-coated 200-mesh nickel grids (Ted Pella Inc., Redding, CA, USA) and allowed to settle for 25 min. The grids were air-dried and observed under a JEM-1400 transmission electron microscope (JEOL) at 100 kV.

### Liquid chromatography-tandem mass spectrometry analysis

After dispensing 200 μL of the extract in a 1.5 mL plastic tube, 50 μL of 10 μM ^15^N5-AMP (internal standard) aqueous solution and 1.0 mL of acetonitrile (ACN) were added. The mixture was centrifuged at 10,000 × *g* for 10 min at 4 °C. The supernatant was transferred to a new tube and dried under vacuum. The residue was dissolved in 100 μL of the solvent A (ACN/10 mM ammonium acetate in H_2_O = 1/9, v/v). Aliquots of the reconstituted solutions were mixed with H_2_O or 100 μM c-di-AMP in H_2_O (1/1, v/v) and filtered through a membrane filter (Millex-LG membrane, 0.45 µm, Millipore). The filtrate was used as a sample for LC–MS/MS. The samples were analyzed using a UPLC system (ACQUITY UPLC I-Class, Waters, Milford, MA, USA) and a triple quadrupole MS (Xevo TQ-S, Waters). A Scherzo SM-C18 MF column (3 µm, 150 mm × 2 mm i.d., Imtakt) was used for separation. The mobile phases A (ACN/10 mM ammonium acetate in H_2_O, 1/9, v/v) and B (ACN/100 mM ammonium acetate in H_2_O, 1/1, v/v) were used for gradient elution as follows: 0–40% B (0–8 min), 40–100% B (8–10 min), 100% B (10–11 min), 100–0% B (11–11.1 min), 0% B (11.1–15 min). The flow rate was set at 0.200 mL/min and the column temperature was maintained at 40 °C. The samples were maintained at 10 °C and 3 µL of each was injected into the column. The conditions for electrospray ionization and MS/MS were set as follows: ion mode, positive; capillary voltage, 3.0 kV; desolvation gas flow, 1000 L/h; cone gas flow, 150 L/h; nebulizer gas flow, 7.0 bar; source temperature, 150 °C; desolvation temperature, 500 °C; and data acquisition mode, MRM. The MRM transitions for c-di-AMP were set as follows: cone voltage, 60 V; precursor ion, m/z 658.7; collision energy, 50 eV; and product ion, m/z 136.1. MRM transitions for ^15^N5-AMP were set as follows: cone voltage, 30 V; precursor ion, m/z 353.1; collision energy, 20 eV; product ion, m/z 141.2. The data were analyzed using MassLynx V4.2. C-di-AMP sodium salt (SML-1231) and adenosine-^15^N5 5′-monophosphate (15N5-AMP) disodium salt (662658) were purchased from Merck KGaA (Darmstadt, Germany).

### Statistical analysis

Data are presented as mean ± SD. The means of multiple groups were compared using analysis of variance (ANOVA), followed by Tukey’s tests using the JSTAT statistical software (version 8.2) and the unpaired Student’s *t*-test. All analyses were performed in a blinded manner without information about the experimental conditions. A value of *P* < 0.05 was considered significant. The animals were randomly assigned to various groups.

### Supplementary Information

**Additional file 1: Figure S1.** Construction of *fliC*-deleted non-AIEC K12 strain. **Figure S2.** Detection of c-di-AMP within in non-AIEC and AIEC.

## Data Availability

All relevant data are within the manuscript and its supporting information.

## Abbreviations

AIEC: Adherent-invasive* Escherichia coli*
CD: Crohn’s disease
c-di-AMP: Cyclic di-AMP
CFU: Colony-forming units
DMEM: Dulbecco's modified Eagle medium
IBD: Inflammatory bowel disease; LB: Luria Bertani
PCR: Polymerase chain reaction
SEM: Scanning electron microscopy
TEM: Transmission electron microscopy
