# Supplementary material for: Cyclic-di-AMP confers an invasive phenotype on Escherichia coli through elongation of flagellin filaments
Source: Gut Pathog. 2024 Jan 24;16:6. doi: 10.1186/s13099-024-00600-4 (PMC10809763; doi:10.1186/s13099-024-00600-4)
Supplement: Supplementary file 1 — Additional file 1: Figure S1. Construction of fliC-deleted non-AIEC K12 strain. Figure S2. Detection of c-di-AMP within in non-AIEC and AIEC. [file 13099_2024_600_MOESM1_ESM.docx]

**Additional Information.**

**Cyclic-di-AMP confers an invasive phenotype on *Escherichia coli* through elongation of flagellin filaments**

**Rika Tanaka^1^, Jin Imai^2^, Eiji Sugiyama^3^, Shogo Tsubaki^4^, Katsuto Hozumi^1^, and Hitoshi Tsugawa^4*^**

**
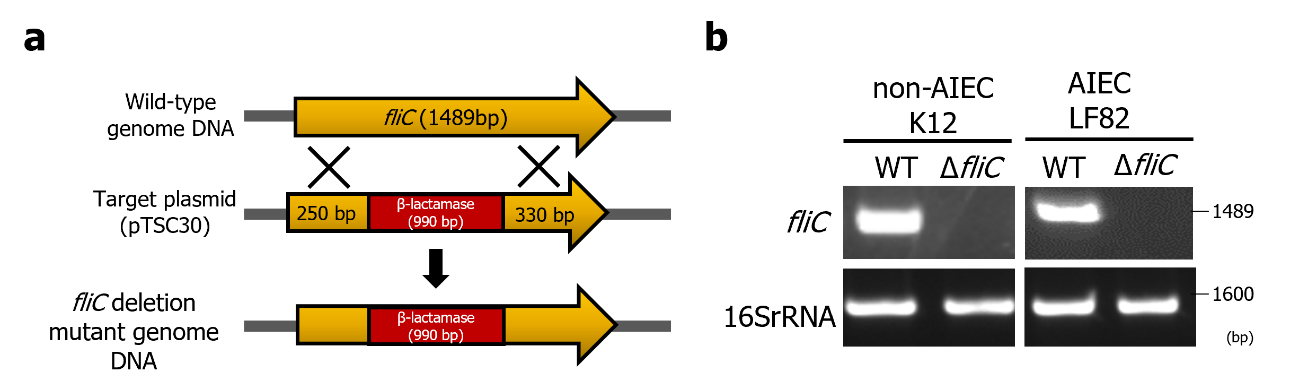
Figure S1. Construction of *fliC*-deleted non-AIEC K12 strain.**

(a)The target-region gene cassette (5′*fliC*–*ampicillin resistance gene*–3′*fliC*) for construction of the *fliC*-deletion mutant was cloned into a temperature-sensitive pTSC30 plasmid. The cassette was inserted into the open reading frame of FliC. The cassette was constructed using the PCR-based overlap extension method. For the overlap extension, the forward primer was the 5′*flic* region forward primer, and the reverse primer was the 3′*flic* region reverse primer. The target-region gene cassette was inserted into the pTSC30 plasmid at the XhoI and BamHI sites using the NEBuilder HiFi DNA Assembly Master Mix. The target pTSC30 plasmid was electroporated into the *E. coli* K12 strains, which were cultured overnight on Luria Bertani agar containing ampicillin (50 μg/mL) at 37 °C to select the resistant colonies. (b) To confirm gene deletion, colony PCR was performed using primers for *fliC*.


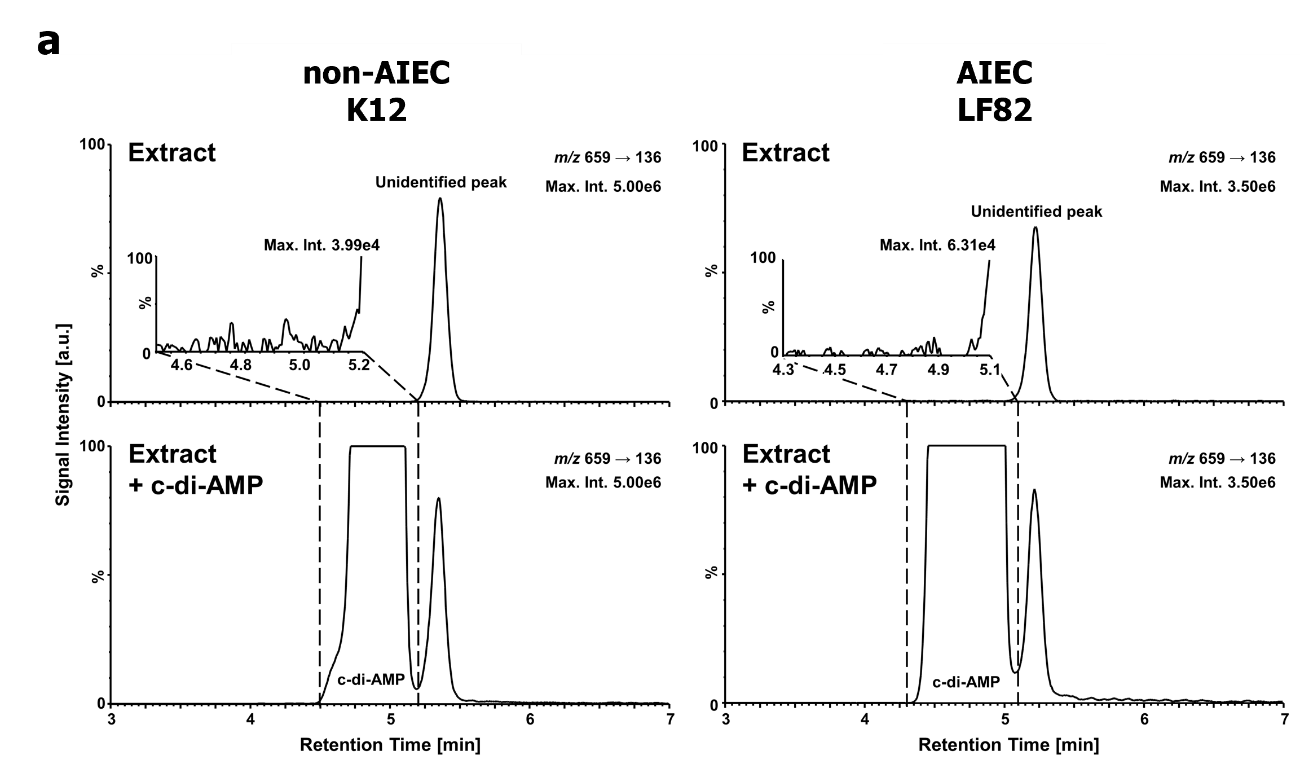


**Figure S2. Detection of c-di-AMP within in non-AIEC and AIEC.**

Liquid chromatography/tandem mass spectrometry (LC-MS/MS) analysis of c-di-AMP in *Escherichia coli* extract. After dispensing 200 μL of the *E. coli* extract in a 1.5 mL plastic tube, 50 μL of 10 μM ^15^N5-AMP (internal standard) aqueous solution and 1.0 mL of acetonitrile were added to the tube. The mixture was centrifuged and the supernatant was transferred to a new tube and dried under vacuum. The residue was dissolved in 100 μL of the solvent A (ACN/10 mM ammonium acetate in H_2_O = 1/9, v/v). Aliquots of the reconstituted solutions were mixed with H_2_O or 100 μM c-di-AMP in H_2_O (1/1, v/v) and were filtered using a membrane filter. The filtrate was used as the sample for LC-MS/MS.
